# Supplementary material for: USP12 downregulation orchestrates a protumourigenic microenvironment and enhances lung tumour resistance to PD-1 blockade
Source: Nat Commun. 2021 Aug 11;12:4852. doi: 10.1038/s41467-021-25032-5 (PMC8357983; doi:10.1038/s41467-021-25032-5)
Supplement: Supplementary file 1 — Supplementary Information [file 41467_2021_25032_MOESM1_ESM.pdf]

Supplementary Information for

USP12 Downregulation Orchestrates a Protumourigenic Microenvironment and Enhances Lung Tumour Resistance to PD-1 Blockade

Zhaojuan Yang[†], Guiqin Xu[†], Boshi Wang[†], Yun Liu, Li Zhang, Tiantian Jing, Ming Tang, Xiaoli Xu, Kun Jiao, Lvzhu Xiang, Yujie Fu, Daoqiang Tang, Xiaoren Zhang, Weilin Jin, Guanglei Zhuang, Xiaojing Zhao[*], Yongzhong Liu[*]

This PDF file includes:

Supplementary Fig. 1 to 7

Supplementary Table 1 to 4

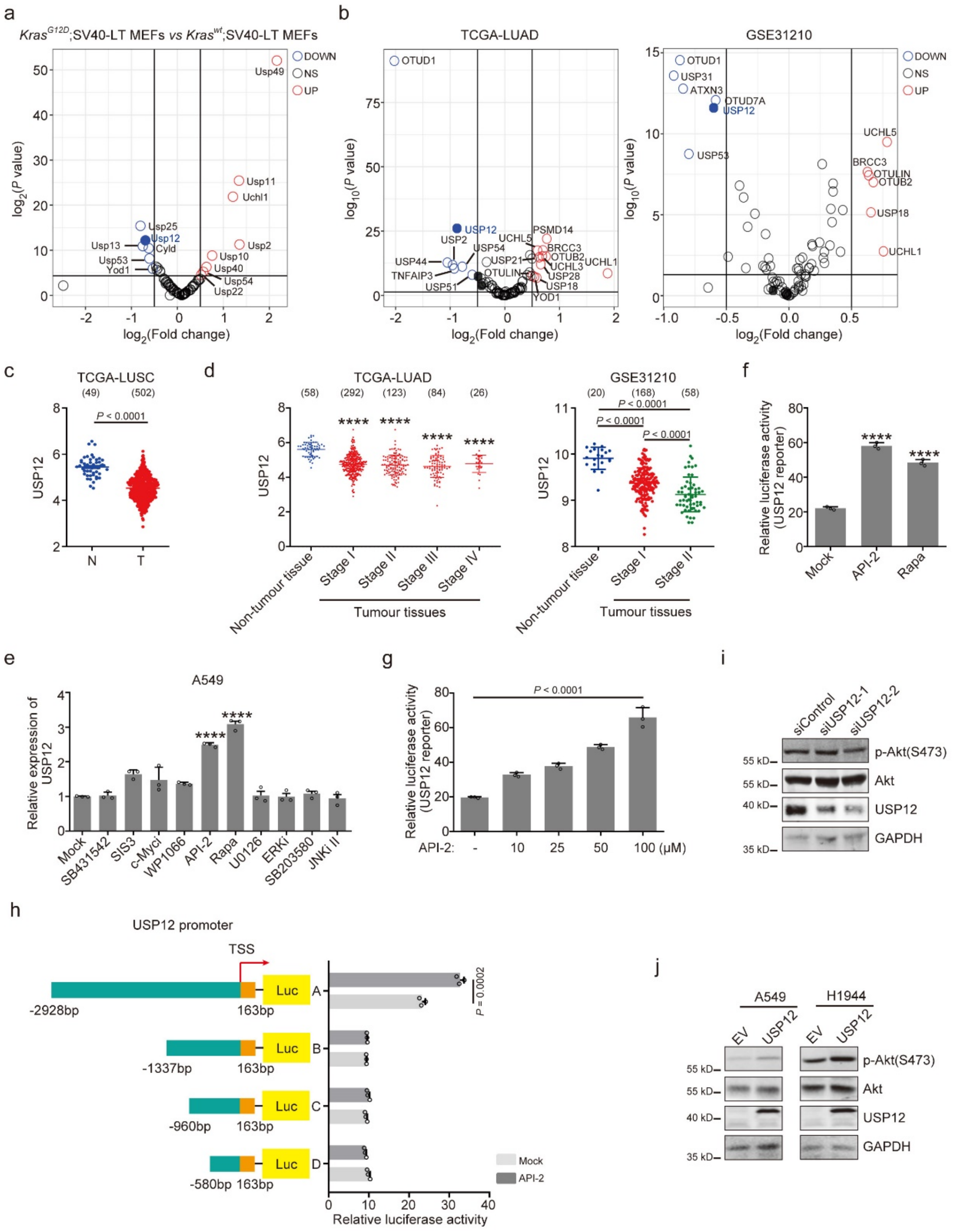

Supplementary Fig. 1 AKT-mTOR signalling downregulates USP12 in lung tumour cells. a Volcano plot showing the differentially expressed DUBs in *Kras*^{G12D};SV40-LT MEFs compared to *Kras*^{wt};SV40-LT MEFs (RNA-seq). Differentially expressed genes were determined with the following criteria: $P \leq 0.05$ and log2 (Fold change) ≥ 0.5 or ≤ -0.5. **b** Volcano plot showing the differentially expressed DUBs in human NSCLC samples compared to non-tumour issued from the TCGA and GSE31210 databases. **c** USP12 levels in LUSC patients from TCGA (mean

± SD). 2-tailed unpaired *t*-test. **d** USP12 levels in NSCLC patients at different tumour stages. The data were obtained from TCGA and GSE31210 databases (mean ± SD). ****$P < 0.0001$ vs. non-tumour tissue by 2-tailed unpaired *t*-test (TCGA-LUAD). One-way ANOVA followed by Tukey's HSD test (GSE31210). **e** Levels of USP12 mRNA in A549 cells after 24 hours of treatment with the indicated inhibitors, SB431542 (10 µM), SIS3 (10 µM), c-Myci (100 µM), WP1066 (10 µM), API-2 (50 µM), Rapamycin (100 nM), U0126 (10 µM), ERKi (10 µM), SB203560 (10 µM), and JNKi II (10 µM) (mean ± SD, $n = 3$ biologically independent samples per group). ****$P < 0.0001$ vs. Mock by 2-tailed unpaired *t*-test. c-Myci: c-Myc inhibitor; Rapa: rapamycin; ERKi: ERK inhibitor; JNKi II: JNK inhibitor II. **f, g** HEK293T cells were transiently transfected with USP12 promoter reporter and Renilla vectors, and then treated with API-2 (100 µM) or Rapamycin (100 nM) (**f**) or API-2 with indicated concentration (**g**) for 24 hours. Relative luciferase activities were analysed (mean ± SD, $n = 3$ biologically independent samples per group). ***$P < 0.001$ vs. Mock by 2-tailed unpaired *t*-test (**f**). One-way ANOVA followed by Tukey's HSD test (**g**). **h** Schematic diagram of serial truncations of the USP12 promoter (left). The numbers represent the end points of each truncated fragment, and the resultant plasmids were named A, B, C, and D. Relative luciferase activities were analysed in indicated HEK293T cells that treated with or without API-2 (25 µM) (mean ± SD, $n = 3$ biologically independent samples per group). 2-tailed unpaired *t*-test. TSS: transcription start site. **i** A549 cells were transiently transfected with USP12-specific siRNAs for 72 hours. Western blotting showing indicated protein levels in cells. **j** Protein levels of p-Akt, Akt, USP12, and GAPDH in A549 or H1944 cells stably transfected with empty control (EV) or USP12. Sample sizes for each group are given in parentheses (**c** and **d**).

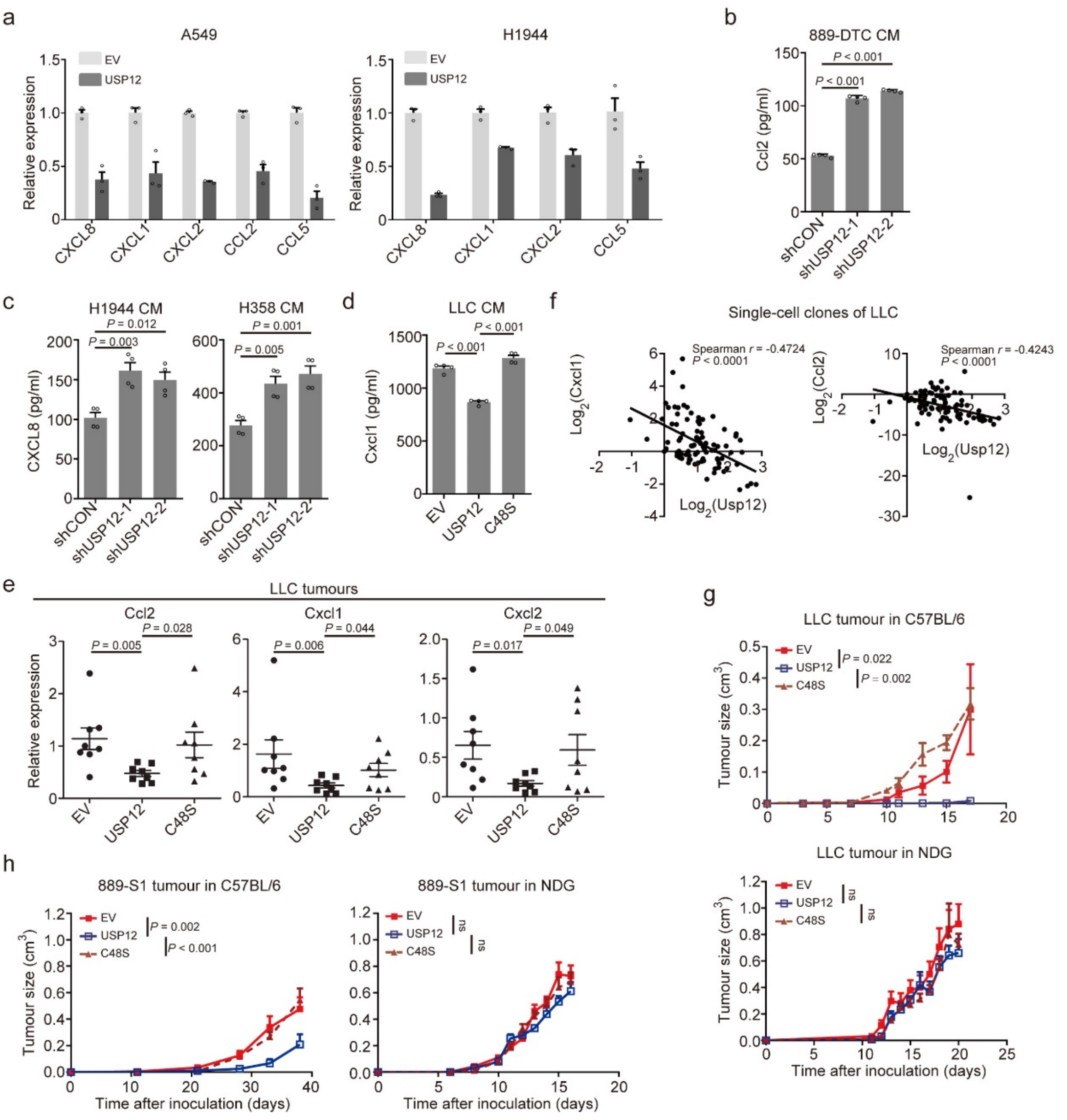

Supplementary Fig. 2 USP12 regulates cytokine expression in tumour cells. a Quantification of CXCL8, CXCL1, CXCL2, CCL2, or CCL5 mRNA levels in indicated cells by qRT-PCR (mean ± SEM, n = 3 biologically independent samples per group). **b** Ccl2 levels 24 hours after knockdown of USP12 expression with USP12 specific shRNAs (shUSP12-1 or shUSP12-2) in conditioned medium (CM) of 889-DTC cells were analysed by ELISA (mean ± SEM, n = 4 biologically independent samples per group). One-way ANOVA followed by Tukey’s HSD test. **c** ELISA analysis of CXCL8 levels in CM of indicated cells after 24 hours of culture (mean ± SEM, n = 4 biologically independent samples per group). Kruskal-Wallis test. **d** ELISA analysis of Cxcl1 levels in CM of LLC cells with or without expression of USP12-WT (USP12) or USP12-C48S (C48S) after 24 hours of culture (mean ± SEM, n = 4 biologically independent samples

per group). Kruskal-Wallis test. **e** Quantification of Cxcl1, Cxcl2, or Ccl2 mRNA levels in LLC tumours by qRT-PCR (mean ± SEM, *n* = 8 biologically independent samples per group). Kruskal-Wallis test. **f** Correlation of mRNA expression between Usp12 and chemokine in LLC single-cell clones by Pearson's correlation analysis (*n* = 90 biologically independent samples). **g, h** Growth of indicated LLC (**g**) and 889-S1 (**h**) tumours in C57BL/6 or B-NDG (NDG) mice (mean ± SEM, *n* = 5 - 8 each group). Two-way ANOVA followed by Bonferroni's multiple comparisons post-test. ns: no significant difference.

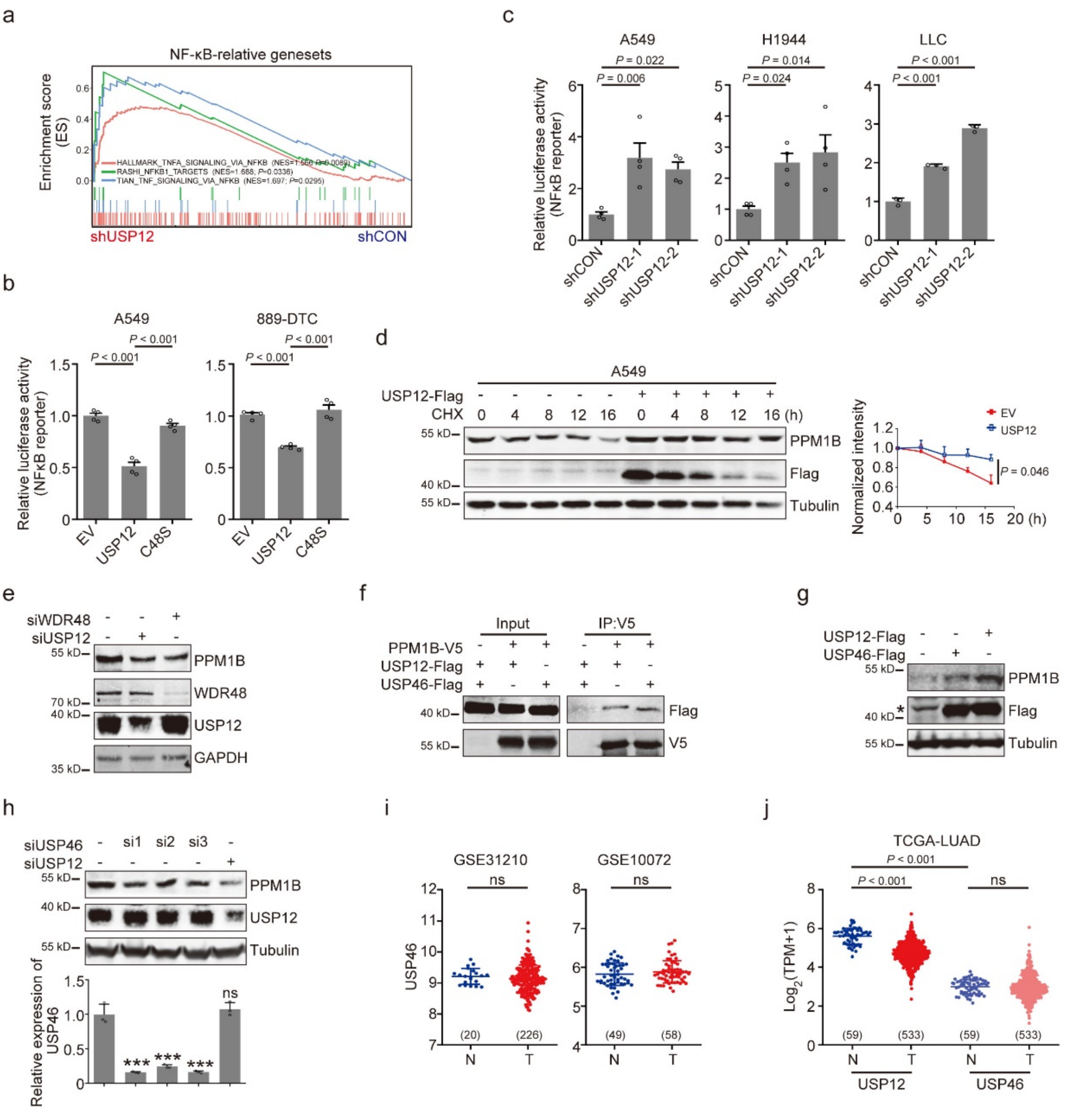

Supplementary Fig. 3 USP12 regulates PPM1B expression and NF-κB signalling activity. **a** GSEA of the transcriptional profiles of USP12-knockdown (shUSP12) H1944 cells and control cells (shCON) with the NF-κB-related signatures. NES: Normalized Enrichment Score. **b** Relative luciferase activities were analysed in A549 or 889-DTC cells stably transfected with empty control, USP12-WT, or USP12-C48S (mean ± SEM, n = 4 biologically independent samples per group). Kruskal-Wallis test. **c** Relative luciferase activities were analysed in A549, H1944 or LLC cells stably transfected with control or two individual USP12 shRNAs (mean ± SEM, n = 3 - 4 biologically independent samples per group). Kruskal-Wallis test. **d** Western blotting analysis of protein expression of PPM1B, USP12, and α-tubulin in A549 cells stably transfected with empty control or USP12 after treatment with Cycloheximide (CHX,

100 μg/ml) for indicated times. A plot of normalized amount of PPM1B protein is shown in right panel (mean ± SEM, *n* = 4 biologically independent samples per group). **e** Western blotting analysis of protein expression of PPM1B, WDR48, USP12, and GAPDH in H358 cells transiently transfected with control siRNAs, WDR48 siRNAs (siWDR48) or USP12 siRNAs (siUSP12) for 72 hours. **f** HEK 293T cells transfected with V5-tagged PPM1B and Flag-tagged USP12 or USP46 were subjected to IP with an anti-V5 antibody, and then immunoblotted with indicated antibodies. **g** Protein levels of PPM1B, USP46, USP12, and α-tubulin in HEK 293T cells transiently transfected with Flag-tagged USP12 or USP46. *: non-specific band. **h** Protein expression of PPM1B, USP12, and α-tubulin in H358 cells transiently transfected with control siRNAs (siControl), USP46 siRNAs (siUSP46) or USP12 siRNAs for 72 hours (top). Quantification of USP46 mRNA levels in indicated cells by qRT-PCR (mean ± SD, *n* = 3 biologically independent samples per group) (bottom). ***$P < 0.001$ vs. siControl by Mann-Whitney U-test. **i** USP46 levels in NSCLC patients from GSE31210 and GSE10072 datasets (mean ± SD). 2-tailed unpaired *t*-test. **j** Levels of USP12 and USP46 in LUAD patients from TCGA (mean ± SD). One-way ANOVA followed by Tukey's HSD test. ns: no significant difference. Sample sizes for each group are given in parentheses (**i** and **j**).

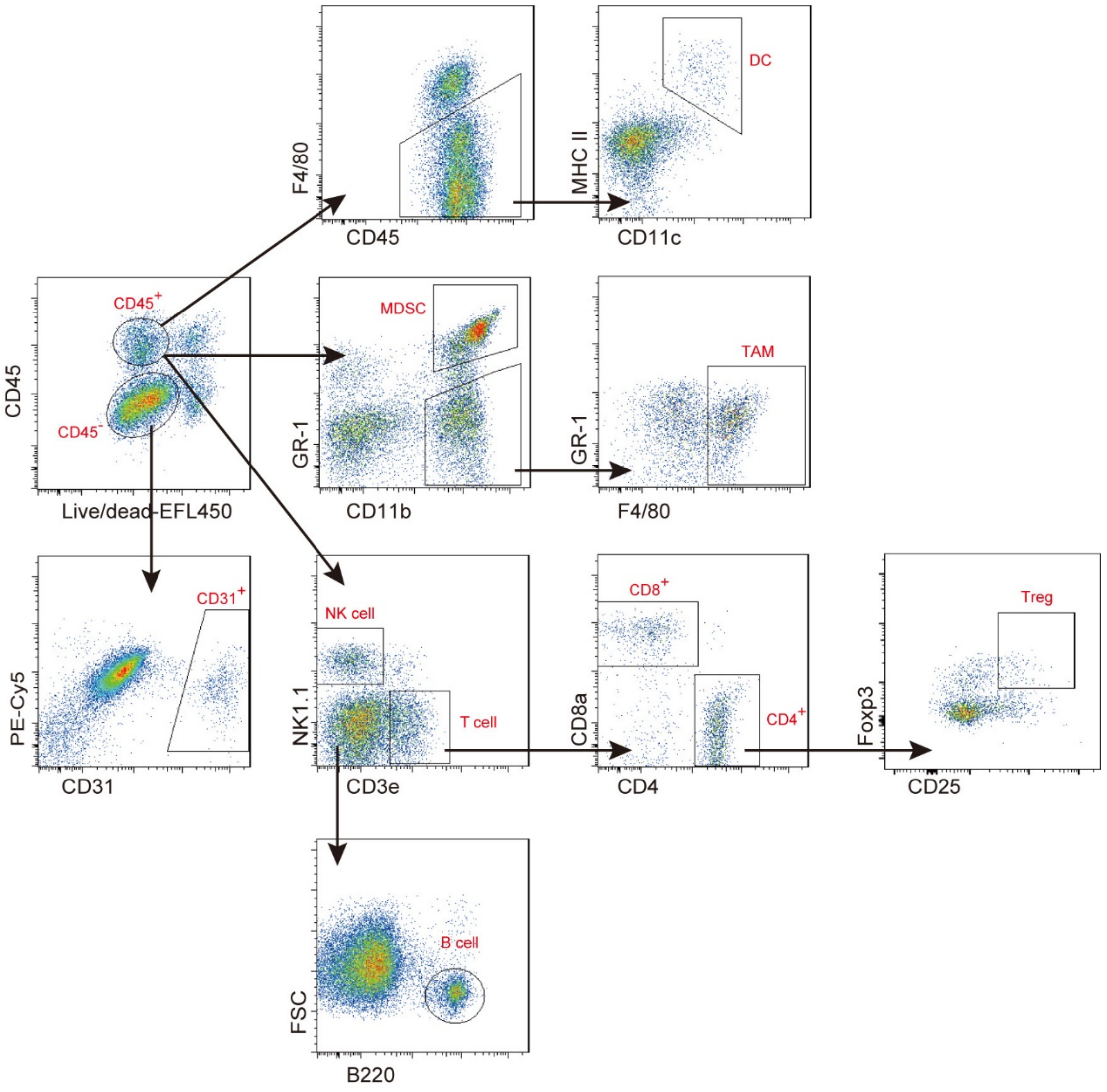

Supplementary Fig. 4 Gating strategy to identify cell subsets in tumour microenvironment. Representative dot plots showing the strategy used to characterize the TME. All subsequent gating used a viability marker. After gating on the $CD45^+$ population, the cells were classified as myeloid-derived suppressor cells (MDSCs, $Gr\text{-}1^+CD11b^+$), tumour-associated macrophages (TAMs, $F4/80^+Gr\text{-}1^-CD11b^+$), dendritic cells (DC, $CD11c^+MHC\ II^+F4/80^-$), natural killer (NK) cells ($NK1.1^+$), B cells ($B220^+$) and T cells ($CD3^+$). T cells were further classified into CD4 ($CD4^+$) and CD8 ($CD8^+$) cells. $CD4^+Foxp3^+CD25^+$ Tregs were a subtype of T cells. The endothelial cells were identified as $CD31^+CD45^-$.

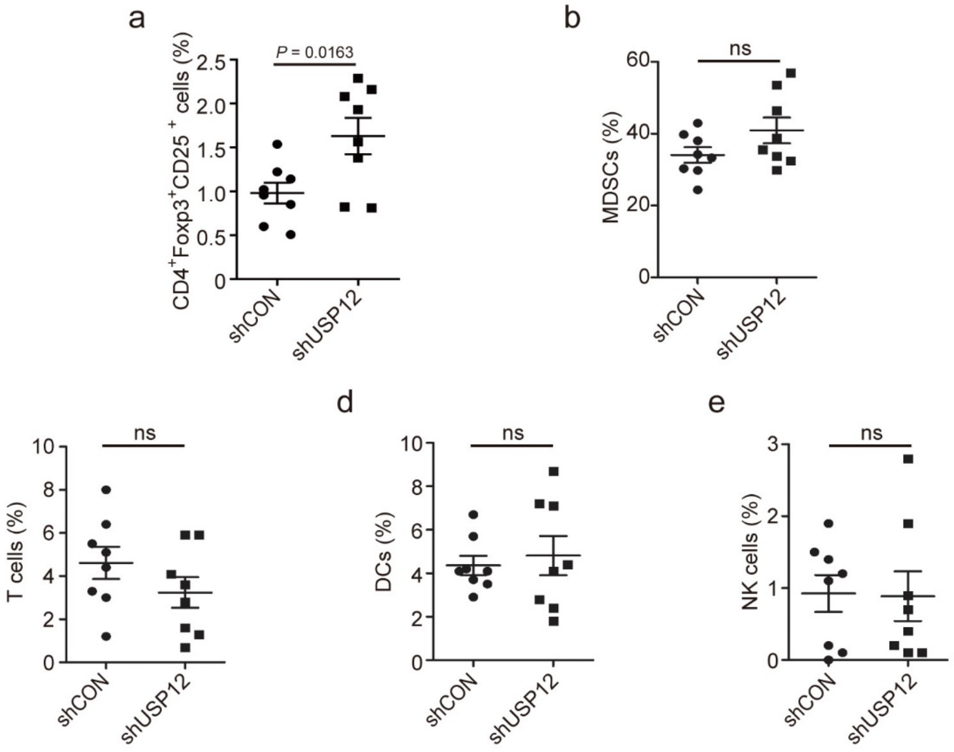

Supplementary Fig. 5 USP12 modulates immune cell subtypes in LLC tumour. Flow cytometric analysis of the proportions of Tregs (**a**), MDSCs (**b**), T cells (**c**), DCs (**d**) and NK cells (**e**) in the LLC-shCON or LLC-shUSP12 tumours (mean ± SEM, *n* = 8 per group). 2-tailed unpaired *t*-test. ns: no significant difference.

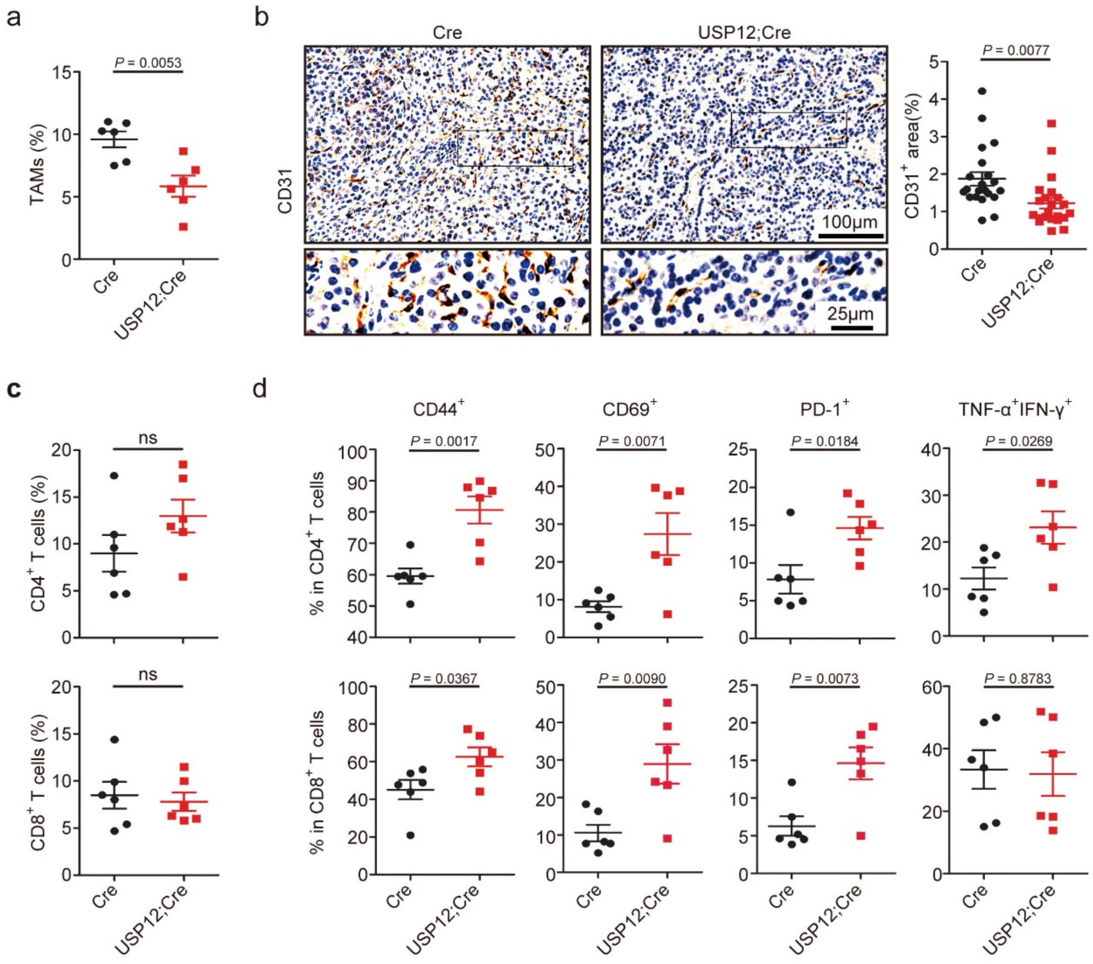

Supplementary Fig. 6 USP12 modulates immune phenotype and angiogenesis in *Kras*^{G12D}-driven lung adenoma mouse model. a Flow cytometric analysis of TAMs in the lungs of *Kras*$^{LSL\text{-}G12D/+}$ mice infected with Cre or USP12;Cre lentiviruses for 8 months (mean ± SEM, n = 6 per group). **b** Representative images (left) and quantification analysis (right) of CD31 immunostaining in lung sections from the Cre or USP12;Cre group (mean ± SEM, n > 20 tumours from >3 mice). **c** Flow cytometric analysis of CD4 and CD8 T cells in lungs of Cre or USP12;Cre group (mean ± SEM, n = 6 per group). **d** Flow cytometric analysis of the expression of CD44, CD69, and PD-1 on CD4 and CD8 T cells, and the proportion of TNF-α$^+$IFN-γ$^+$ cells gated on CD4 and CD8 T cells in the lungs of Cre or USP12;Cre group (mean ± SEM, n = 6 per group). 2-tailed unpaired *t*-test. ns: no significant difference.

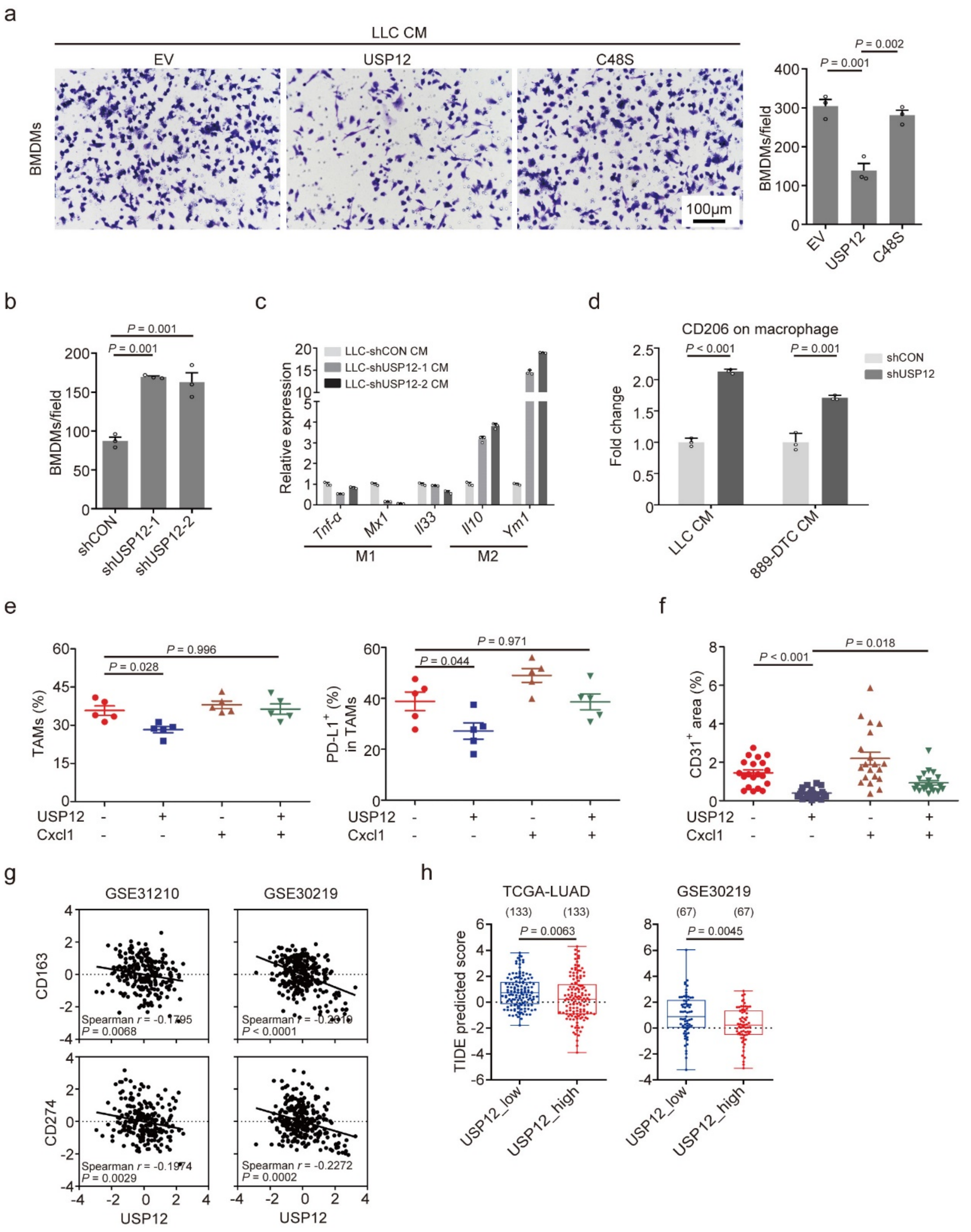

Supplementary Fig. 7 USP12 inhibits recruitment and polarization of macrophage. **a** Representative images (left) and quantification (right) of the migration of BMDMs cultured in CM from LLC cells (mean ± SEM, n = 3 fields of view). One-way ANOVA followed by Tukey's HSD test. **b** Quantification of the migration of BMDMs cultured in CM from indicated LLC cells (mean ± SEM, n = 3 fields of view). One-way ANOVA followed by Tukey's HSD test. **c** qRT-PCR analysis of the mRNA levels of Tnf-α, Mx1,

Il33, Il10, and Ym1 in BMDMs stimulated with CM from LLC cells for 48 hours (mean ± SD, n = 3 per group). **d** Flow cytometric analysis of the expression of CD206 on BMDMs stimulated with CM from indicated cells for 48 hours. Data are presented as fold change in shUSP12 cells vs. shCON cells (mean ± SD, n = 3 per group). 2-tailed unpaired t-test. **e** The proportion of TAMs and PD-L1 expression on TAMs in LLC tumours (mean ± SEM, n = 5 per group). One-way ANOVA followed by Tukey's HSD test. **f** Quantification analysis of CD31 immunostaining in LLC tumours (mean ± SEM, n = 20 fields of view from > 3 mice). One-way ANOVA followed by Tukey's HSD test. **g** USP12 negatively correlates with CD163 and CD274 in NSCLC by Pearson's correlation analysis. **h** Boxplot of TIDE prediction score in USP12_low (bottom 25%) and USP12_high (top 25%) NSCLC group. Sample sizes for each group are given in parentheses. The centre mark represents median, and whiskers show minimum/maximum values. 2-tailed unpaired t-test.

Supplementary Table 1. List of Primers used for gene cloning

Genes	Primers (5'-3')
Human	
USP12	ATGGAAATCCTAATGACAGTCTCC
	TCAGTCCCGAGACTGATAGAAAAGG
USP46	ATGACTGTCCGAAACATC
	CTCTCTTGACTGATAGAATA
PPM1B	ATGGGTGCATTTTTGGATAAACCCAAAACTG
	TATTTTTTCACCACTCATCTTTGTCCCTGC
Mouse	
Cxcl1	ATGATCCCAGCCACCCGCTC
	CTTGGGGACACCTTTTAGC
Ccl2	ATGCAGGTCCCTGTCATGC
	GTTCACTGTCACACTGGTC

Supplementary Table 2. Primers used for construction of pGL3-basic-based vectors

Primer names	Primers (5'-3')
Human	
USP12-PMT-163-R	CGCCATCTTCCACCCAATCAC
USP12-PMT--2928-F	TCCAAGCTCAGGGTGCAAAA
USP12-PMT-163-ha-R	TAGATCGCAGATCTCGAG CGCCATCTTCCACCCAATCAC
USP12-PMT--580-ha-F	TACGCGTGCTAGCCCGGG CAAGTGCTTGGGACGCATAC
USP12-PMT--960-ha-F	TACGCGTGCTAGCCCGGG AACTACCCATGCCAGTAGCC
USP12-PMT--1337-ha-F	TACGCGTGCTAGCCCGGG AGATCCAGTCCGGCTACCAT
USP12-PMT--2928-ha-F	TACGCGTGCTAGCCCGGG TCCAAGCTCAGGGTGCAA

Supplementary Table 3. List of Primers used for RT-PCR

Genes	Primers (5'-3')
human	
CXCL8	ACCACCGGAAGGAACCATCTCAC
	AGCACTCCTTGGCAAAACTGCAC
CXCL1	GGCGGAAAGCTTGCCTCAATCCT
	AACAGCCACCAGTGAGCTTCCTC
CXCL2	TTCACAGTGTGTGGTCAACAT
	TCTCTGCTCTAACACAGAGGGA
CCL2	TCAAACTGAAGCTCGCACTCT
	GGGGCATTGATTGCATCTGG
CCL5	ATGACTCCCGGCTGAACAAG
	CAGGTTCAAGGACTCTCCATCC
PPM1B	TGTGTGCAATTGGGTAGTGG
	TCACCGCTTCATCTGAGACC
USP12	TCCGGTCAATGAGCACTATTT
	CCTAGGTTGACTCTTATACGCA
USP46	TCCATCTGTAATATGGGCACCAA
	ACGGCAGAAGTACAATGCCT
GAPDH	TCCTGTTCGACAGTCAGCCGCA
	ACCAGGCGCCCAATACGACCA
Mouse	
Cxcl1	ACTCAAGAATGGTCGCGAGG
	GTGCCATCAGAGCAGTCTGT
Cxcl2	CACTCTCAAGGGCGGTCAA
	CACATCAGGTACGATCCAGGC
Ccl2	AGCATCCACGTGTTGGCTC
	CCAGCCTACTCATTGGGATCA
Vegf	ACTGGACCCTGGCTTTACTG
	GCAGTAGCTTCGCTGGTAGA
Nrp1	CACAGTGGCACAGGTGATGA
	ACCGTATGTCGGGAACTCTG
Mmp9	GCGTCATTCGCGTGGATAAG
	TGGAAACTCACACGCCAGAA
Mmp2	TGTGCGACCACAACCAACTA
	GTCCTGAGAGTGTTCCAGCC
Usp12	GGTCAACGAGCACTATTTTGGA
	TCTGGGCTGACTTTTGTATGC
Gapdh	AGGTCGGTGTGAACGGATTTG
	TGTAGACCATGTAGTTGAGGTCA

Supplementary Table 4. Characteristics of patients with NSCLC for experimental analyses.

Patient characteristics	No. of cases (%)
Age (years)	
≦ 60	10 (33%)
> 60	20 (67%)
Gender	
Male	14 (47%)
Female	16 (53%)
Histopathological subtype	
ADC	24 (80%)
SCC	4 (13%)
ASC	2 (7%)

ADC, adenocarcinoma; SCC, squamous cell carcinoma; ASC, adenosquamous carcinoma.
